# Predicting Long Noncoding RNA and Protein Interactions Using Heterogeneous Network Model

**DOI:** 10.1155/2015/671950

**Published:** 2015-12-29

**Authors:** Ao Li, Mengqu Ge, Yao Zhang, Chen Peng, Minghui Wang

**Affiliations:** ^1^School of Information Science and Technology, University of Science and Technology of China, 443 Huangshan Road, Hefei 230027, China; ^2^Centers for Biomedical Engineering, University of Science and Technology of China, 443 Huangshan Road, Hefei 230027, China

## Abstract

Recent study shows that long noncoding RNAs (lncRNAs) are participating in diverse biological processes and complex diseases. However, at present the functions of lncRNAs are still rarely known. In this study, we propose a network-based computational method, which is called lncRNA-protein interaction prediction based on Heterogeneous Network Model (LPIHN), to predict the potential lncRNA-protein interactions. First, we construct a heterogeneous network by integrating the lncRNA-lncRNA similarity network, lncRNA-protein interaction network, and protein-protein interaction (PPI) network. Then, a random walk with restart is implemented on the heterogeneous network to infer novel lncRNA-protein interactions. The leave-one-out cross validation test shows that our approach can achieve an AUC value of 96.0%. Some lncRNA-protein interactions predicted by our method have been confirmed in recent research or database, indicating the efficiency of LPIHN to predict novel lncRNA-protein interactions.

## 1. Introduction

Long noncoding RNAs (lncRNAs), a class of important non-protein coding transcripts with lengths more than 200 nucleotides [1], have gained wide attention recently, and a large number of lncRNAs have been discovered by analysis of chromatin-state maps [2] and full-length complementary DNA (cDNA) [3] based on RNA-seq data [4]. Recent researches show that lncRNAs play critical roles in complex cellular processes, such as epigenetic regulation of gene expression [5–9], chromatin modification [10], and cell differentiation. Moreover, studies show that a number of lncRNAs are implicated in a range of human diseases [11–13]. Hence, uncovering the functions of lncRNAs is of great importance in understanding the mechanisms of biological processes.

Generally, almost all of the lncRNAs function through interactions with corresponding RNA binding proteins [14–16]. In turn, RNA binding proteins can interact with different lncRNAs to regulate diverse cellular processes [17, 18]. Thus, identifying the potential lncRNA-protein interactions is critical to understand the functions of lncRNAs. Since experimental detection of unknown lncRNA-protein interactions is time consuming and costly, some computational approaches have been proposed for lncRNA-protein interaction prediction. In 2011, CatRAPID was developed by Bellucci et al. [5], in which lncRNA-protein pairs are encoded into feature vectors and scored by using matrix computation. In the same year, a method named RPIseq was introduced by Muppirala et al. [19] using random forest (RF) and support vector machines (SVM) classifiers to predict lncRNA-protein interaction and RPIseq only uses the sequence information of lncRNAs and proteins. In 2013, Lu et al. [20] introduced a method named lncPro, which predicts lncRNA-protein interactions by using scores yielded by amino acid and nucleotide sequences and Fisher's linear discriminant method.

In this paper, we introduce a network-based method, lncRNA-protein interaction prediction based on Heterogeneous Network Model (LPIHN), to predict the interactions between lncRNAs and proteins. First, we construct a heterogeneous network with the use of protein-protein interaction (PPI), lncRNAs expression similarity, and known lncRNA-protein interactions. Then, a random walk with restart is implemented on the heterogeneous network to infer novel lncRNA-protein interactions. We compare the performance with two network-based methods including PRIoritizatioN and Complex Elucidation (PRINCE) [21] and the random walk based method (RWR) [22]. In the leave-one-out cross validation (LOOCV) test we implement, LPIHN outperforms PRINCE and RWR by a significant margin. Moreover, we identify several lncRNA-protein interactions that are supported by evidence in recent literature or database, which shows the practical value of our method.

## 2. Materials and Methods

### 2.1. lncRNA-Protein Interactions

The development of bioinformatics and experimental technologies has made the global lncRNA-protein interaction network available. NPinter (http://www.bioinfo.org/NPInter/) is the up-to-data database that has collected experimentally validated interactions between noncoding RNAs (ncRNAs) and other biomoleculars [23]. The research done by Shang et al. [24] has extracted lncRNA-protein interactions from NPinter and made detailed and comprehensive analysis about the lncRNA-protein network.

In this paper, we download known ncRNA-protein interaction dataset from Npinter 2.0 database in November 2013 and then filter the ncRNAs and their interaction proteins, by restricting the organism and the type of ncRNAs to “Homo sapiens” and “NONCODE,” respectively. Then we further select the lncRNAs from these ncRNAs according to human lncRNA dataset from NONCODE 4.0 database [25] and map the lncRNA ID and protein ID into NONCODE 4.0 ID and string ID separately. *I* is defined as the adjacency matrix of lncRNA-protein interactions, in which *I*(*i*, *j*) is 1 if there is an interaction between protein *i* and lncRNA *j*, otherwise 0.

### 2.2. lncRNA Expression Similarity

The lncRNA expression profiles are obtained from NONCODE 4.0 database, including the expression profiles of 89,369 lncRNA in 24 human tissues or cell types. Then Pearson correlation coefficient (PCC) [26–31] between the expression profiles of each pair of lncRNAs is calculated as the lncRNA expression similarity. We define *X* = {*x*_1_, *x*_2_,…, *x*_24_} and *Y* = {*y*_1_, *y*_2_,…, *y*_24_} as two expression profiles of lncRNA *i* and *j*, respectively, which contain expression value of 24 human tissues or cell types. The expression similarity matrix of the lncRNAs SL can be calculated as

(1) $$\begin{array}{c} \text{SL}\left(\;i,j\;\right)=\left|\;\frac{\mathrm{cov}\left(X,Y\right)}{\sigma_{X}\sigma_{Y}}\;\right|, \end{array}$$

where SL(*i*, *j*) in row *i* and column *j* represents the absolute value of PCC between lncRNA *i* and *j*, cov(*X*, *Y*) is the covariance of *X* and *Y*, and *σ*_*X*_ and *σ*_*Y*_ are the standard deviation of *X* and *Y*, respectively. Calculate PCC between the expression profiles of each pair of nodes which is widely used in bioinformatics research. Hence, the similarity calculated based on the expression data of lncRNA can obtain reliable performance.

### 2.3. Protein-Protein Interactions

We obtain PPI data from STRING 9.1 database [32], which contains weighted protein interactions derived from computational prediction methods, high-throughput experiments, and text mining. Then, we remove the redundant PPI data, resulting in 804 PPI data and corresponding interaction scores according to the known lncRNA-protein dataset, and all PPI pairs are treated as identically reliable. The symmetric matrix SP is defined as the interaction matrix, in which SP_*ij*_ is the interaction score of vertices *i* and *j*. Formally, define a diagonal matrix *M*, in which *M*(*j*, *j*) is the sum of row *j* of SP; the normalization of SP is defined by the following function:

(2) $$\begin{array}{c} {\text{SP}}_{ij}^{\prime }=\frac{{\text{SP}}_{ij}}{\sqrt{M\left(i,i\right)M\left(j,j\right)}}, \end{array}$$

where SP′ is a normalized form of SP.

### 2.4. The Heterogeneous Network

**Figure 1:**
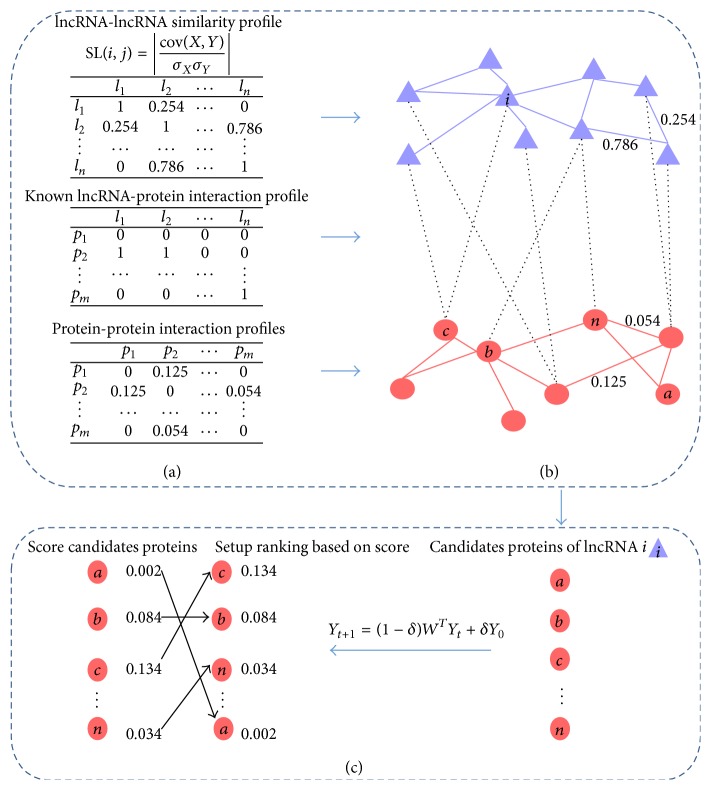
A simple example of the procedure of predicting lncRNA-protein interactions with LPIHN. (a) The lncRNA-lncRNA similarity matrix is calculated by using the expression profiles of lncRNAs to calculate the PCC of each pair of lncRNAs. The profile of known lncRNA-protein interactions is obtained where the value of *p*_*i*_ and *l*_*j*_ is 1 if there exists interaction between lncRNA *l*_*j*_ and protein *p*_*i*_, otherwise 0. The PPI profile is obtained based on the normalized score of PPI. (b) The upper purple network is the lncRNA-lncRNA similarity network, the lower red network is the PPI network, and both of them are constructed based on the corresponding profile in (a). The heterogeneous network is constructed by connecting the lncRNA-lncRNA similarity network and PPI network together with the known lncRNA-protein interaction network. Purple triangles indicate lncRNAs, red circles proteins, purple edges lncRNA-lncRNA similarities, red edges protein-protein interactions, and black dotted edges known lncRNA-protein interactions. (c) Our method assigns a score to each of the candidate proteins of a query lncRNA, with the random walk with restart implemented on the heterogeneous network. The candidate proteins are ranked based on the score.

*G*_1_(*L*, *E*_1_, SL) is defined as the lncRNA-lncRNA similarity network, in which *L* = {*l*_1_, *l*_2_,…, *l*_*n*_} represents the set of *n* lncRNAs, *E*_1_ = {*e*_1_, *e*_2_,…, *e*_*k*_} represents sets of edges between vertices, *l*_*i*_ and *l*_*j*_ are connected if the similarity SL_*ij*_ calculated by PCC between *l*_*i*_ and *l*_*j*_ is more than 0. The PPI network *G*_2_(*P*, *E*_2_, SP′) can be constructed analogously, and vertices set *P* = {*p*_1_, *p*_2_,…, *p*_*m*_} represents the set of *m* proteins. *E*_2_ represents sets of edges between proteins; *p*_*i*_ and *p*_*j*_ will be connected if the normalized interaction score SP_*ij*_′ between vertices *p*_*i*_ and *p*_*j*_ is more than 0. In the lncRNA-protein network, *p*_*i*_ and *l*_*j*_ are connected if *I*(*i*, *j*) is 1. lncRNA-protein heterogeneous network is constructed by connecting the aforementioned lncRNA-lncRNA similarity network and PPI network together with lncRNA-protein interaction network (Figure 1(b)). Then, a random walk with restart will be implemented on the network.

### 2.5. LPIHN Method

LPIHN is proposed to score proteins for each lncRNA by implementing random walk with restart on the heterogeneous network, based on the assumption that similar lncRNAs tend to exhibit similar interaction patterns with proteins. The procedure of random walk with restart is that an iterative walker starts at a source node with an initial probability and transits to a randomly selected direct neighbor; in the process of random walking, the walker can restart at source node with some probability in every time step. Hence, when implementing the random walk on the heterogeneous network, the initial probability, transition matrix, and restart probability should be determined based on the information supplied by the heterogeneous network. In the procedure of predicting the potential proteins for lncRNA *l*_*i*_, let *Y*_0_ represent the initial probability of walker starting at each node, where *l*_*i*_ and the proteins that are known to interact with *l*_*i*_ are assigned positive values and the remaining nodes are assigned zero. This assignment suggests that the random walker starts at *l*_*i*_ or the proteins interact with *l*_*i*_. Let *Y*_*t*_ represent the relevance of *l*_*i*_ to all other nodes, in which the *j*th element indicating the probability of the random walker is found at node *j* at step *t*. *Y*_*t*+1_ can be decided by the following iterative equation:

(3) $$\begin{array}{c} Y_{t+1}=\left(\;1-\delta \;\right)W^{T}Y_{t}+\delta Y_{0}, \end{array}$$

where *δ* ∈ (0,1) represents the restart probability of random walk. *W* is the transition matrix and *Y*_0_ is the initial probability of the random walk. All of them are detailed later.

Given a query lncRNA *l*_*i*_, *l*_*i*_ is the seed node in the lncRNA network, the probability of vertex *l*_*i*_ is 1, and other vertices in the lncRNA network are assigned 0, forming the initial probability of lncRNA network *v*_0_. If protein *p*_*j*_ interacts with lncRNA *l*_*i*_, then *p*_*j*_ is the seed node in the protein network. The initial probability vector of protein network *u*_0_ is formed by assigning equal probabilities to the protein seed nodes, under the condition that the sum is equal to 1. For the heterogeneous network, the initial probability is

(4) $$\begin{array}{c} Y_{0}=\left[\begin{array}{c} \left(1-\beta \right)u_{0}\\ \beta v_{0} \end{array}\right]. \end{array}$$

We use the parameter *β* ∈ (0,1) to weight the importance of lncRNA network and protein network. If *β* = 0.5, lncRNA-lncRNA similarity network and PPI network are equally weighted. If *β* < 0.5, the random walk tends to return to the protein network.

In order to implement random walk on the heterogeneous network, the transition matrix *W* must be defined. We define $W=\left[\begin{array}{cc} W_{P} & W_{PL}\\ W_{LP} & W_{L} \end{array}\right]$ as the transition matrix, where *W*_*P*_ and *W*_*L*_ are the subnetwork transition matrix showing the probability of the random walker transiting from one protein (lncRNA) to another protein (lncRNA) in the process of random walk. *W*_PL_ indicates the probability of the random walker transiting from protein network to lncRNA network and *W*_LP_ indicates the movement from lncRNA network to protein network. In the process of transition, we define *γ* as the probability of random walker transiting from protein network to lncRNA network and vice versa. *W* is defined as follows.

The probability of the random walker transiting from protein *p*_*i*_ to *p*_*j*_ is defined as

(5) WPi,jppj ∣ pi=SP′i,j∑jSP′i,jif  ∑kIi,k=01−γSP′i,j∑jSP′i,jotherwise.

∑_*k*_ *I*(*i*, *k*) = 0 means that *p*_*i*_ only connects to proteins, and the walker can only transit randomly to the direct neighbor protein in the PPI network next step. Otherwise, the walker can transit to the lncRNA-lncRNA network from *p*_*i*_ with probability *γ*; under that condition, the probability of *p*_*i*_ transiting to *p*_*j*_ should multiply 1 − *γ*.

Analogously, the probability from lncRNA *l*_*i*_ to *l*_*j*_ can be defined as

(6) WLi,jplj ∣ li=SLi,j∑jSLi,jif  ∑kIk,i=01−γSLi,j∑jSLi,jotherwise.

The probability from protein *p*_*i*_ to lncRNA *l*_*j*_ is defined as

(7) WPLi,jplj ∣ pi=γIi,j∑jIi,jif  ∑kIi,k≠00otherwise.

∑_*k*_ *I*(*i*, *k*) ≠ 0 means that *p*_*i*_ connects to at least one lncRNA, and the walker can transit to lncRNA-lncRNA network from *p*_*i*_ with probability *γ*; under that condition, we can further calculate the probability of *p*_*i*_ transiting to *l*_*j*_. Otherwise, the probability of *p*_*i*_ transiting to *l*_*j*_ is 0.

The probability from lncRNA *l*_*i*_ to protein *p*_*j*_ can be defined in a similar manner as

(8) WLPi,jppj ∣ li=γIj,i∑jIi,jif  ∑kIk,i≠00otherwise.

As the initial probability *Y*_0_ and the transition matrix *W* are defined, the random walk with restart can be implemented on the heterogeneous network. After several iterations, the change between *Y*_*t*_ and *Y*_*t*+1_ is less than 10^−10^, indicating that a stable probability $Y_{\infty }=\left[\begin{array}{c} (1-\beta )u_{\infty }\\ \beta v_{\infty } \end{array}\right]$ is obtained.

### 2.6. Leave-One-Out Cross Validation Test

We implement a LOOCV procedure to test the performance of LPIHN. With each cross validation trial, each known lncRNA-protein interaction is used as test data and the rest are taken as training dataset. Then the method is evaluated by successfully reconstructing the hidden interaction.

ROC curves are used to evaluate the performance of the method; for a rank threshold *s*, sensitivity (Sn) and specificity (Sp) are defined as follows:

(9) Sn=TPTP+FN,Sp=TNTN+FP.

TN and TP represent the number of negative sites and positive sites that are correctly predicted. FN and FP represent the number of positive sites and negative sites that are wrongly predicted. We plot Sn versus 1 − Sp at different thresholds separating the prediction [33], which is the ROC curve. We calculate the AUC, which is the area under the ROC curve. Meanwhile, some common used measurements, namely, accuracy (Acc), precision (Pre), and Matthew's correlation coefficient (MCC), are calculated as follows:

(10) Acc=TN+TPTN+TP+FN+FP,Pre=TPTP+FP,MCC=TP×TN−FP×FNTP+FN×TP+FP×TN+FN×TN+FP.

We also use the precision versus recall and fold enrichment to measure the performance. For lncRNA *l*_*i*_, the top *k* ranked proteins are considered to interact with *l*_*i*_ in our method. Precision means the fraction of true lncRNA-protein interactions that ranked among the top *k* in the procedure of cross validation. Recall means the fraction of hidden interaction is reconstructed that ranked within top *k*. In this paper, another measure for the evaluation of the method is fold enrichment. For a query lncRNA, the number of its candidate proteins is defined as *N*, the test protein is ranked *n* in the candidate protein set, and the fold enrichment can be calculated by the following formula: fold enrichment = *N*/2/*n*, and here we use the average fold enrichment of all test data for assessment.

## 3. Results

### 3.1. Comparison with Other Network-Based Methods

We compare the performance of LPIHN with other two network-based methods as follows: PRINCE [21] and RWR [22]. In RWR method, for one lncRNA, at least two proteins are required to perform LOOCV. Therefore, we only consider lncRNAs that are interacting with at least two proteins. After the preprocessing, we obtain 1,113 lncRNAs and 96 proteins. And 4,870 lncRNA-protein interactions are regarded as gold-standard dataset to be used in cross validation. Then, LOOCV is implemented to evaluate the performance of these methods. According to previous research [34], we set *β* = 0.5, *γ* = 0.5 here and fix *δ* to 0.3, as it has been reported that the restart probability *δ* has a very slight effect on the result [22, 35].

**Figure 2:**
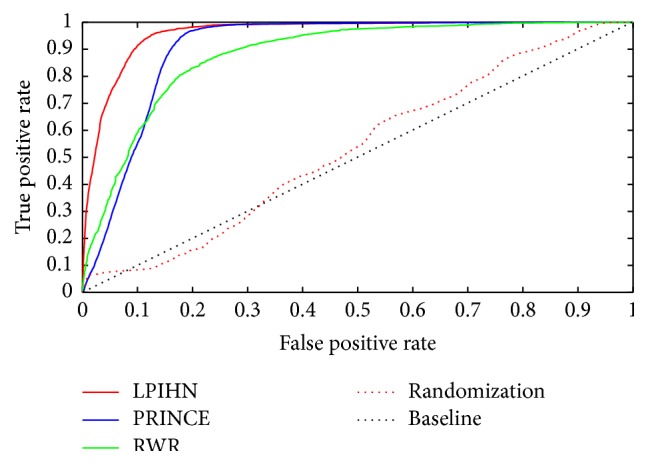
ROC curves of lncRNA-protein interaction predictions by different methods. The red, blue, and green curves are the ROC curves of LPIHN, PRINCE, and RWR, respectively. The red dotted line represents the ROC curve of LPIHN over randomized lncRNA-protein network. The largest area under the curve (AUC) indicates the best performance of potential lncRNA-protein interaction prediction.

The ROC curves of LPIHN, PRINCE, and RWR are plotted in Figure 2, which clearly shows that the ROC curve of LPIHN is consistently above the other two methods. From Table 1, we can see that LPIHN achieves an AUC of 96.0%. The result is higher than PRINCE and RWR, which achieves AUC of 90.6% and 88.1%, respectively. This phenomenon indicates that the performance of LPIHN is better than PRINCE and RWR. To further evaluate that the prediction obtained by our method is not generated by chance, we perform the LOOCV test on random lncRNA-protein interaction network. The lncRNA-protein interaction network is randomized for 1000 times, which means we select seed proteins randomly for each lncRNA. The AUC value of randomization process is 53.0%, which is much lower than AUCs of other three methods. This indicates that our method can discover potential lncRNA-protein interactions. Besides AUC value, we also compare the Sn and Sp  of these methods (Table 1). When the value of Sp is 99.0%, LPIHN achieves a Sn of 35.0%, which is 30.0% and 20.9% higher than other two methods, respectively. When the value of Sp decreases to 90.0%, the Sn value of LPIHN increases to 91.4%, which is 36.3% and 32.2% higher than PRINCE and RWR, respectively. Moreover, we download the update ncRNA-protein dataset (Npinter 3.0) and extract lncRNA-protein interactions according to the 1,113 lncRNAs from Npinter 2.0 dataset. The number of known lncRNA-protein interactions is increased from 4870 to 10232. The ROC curve and AUC value LPIHN, PRINCE, and RWR on the new dataset are displayed in Figure S1 (see Supplementary Material available online at http://dx.doi.org/10.1155/2015/671950), which indicates the same good performance of our method.

**Figure 3:**
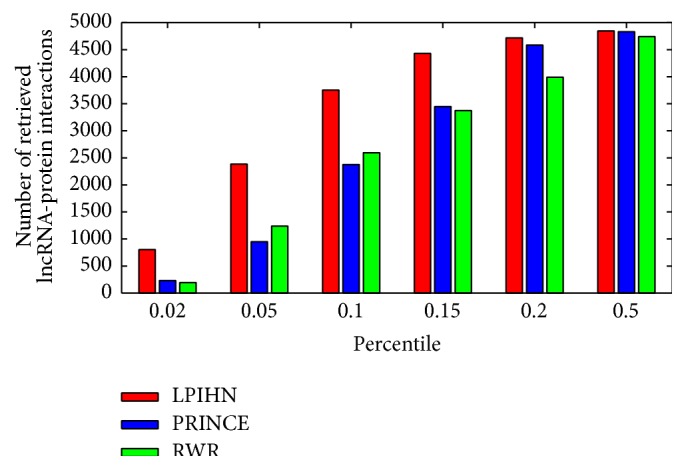
The number of correctly retrieved lncRNA-protein interactions out of total 4,870 true interactions for different percentiles. The red, blue, and green bars represent LPIHN, PRINCE, and RWR, respectively.

In addition, the numbers of retrieved lncRNA-protein interactions in different percentiles are shown in Figure 3, in which the top-ranked reconstructions are especially important because of the lower number of false positives. The result shows that among the top 2% true lncRNA-protein interactions, 802 interactions are predicted successfully based on LPIHN. However, only 229 and 192 interactions are among the top 2% predictions based on PRINCE and RWR, respectively. Besides, LPIHN also achieves a higher number in all the other percentiles than PRINCE and RWR in this comparison.

**Figure 4:**
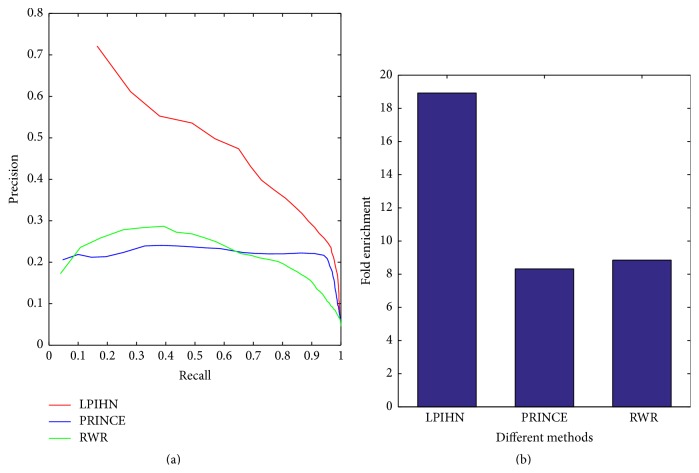
Comparison of different methods in terms of precision versus recall and fold enrichment. (a) Precision versus recall of three different methods when considering the top *k* proteins for different values of threshold *k*. The red, blue, and green lines represent LPIHN, PRINCE, and RWR, respectively. Comparison between LPIHN, PRINCE, and RWR in terms of precision versus recall shows the performance advantage of our method. (b) Enrichment analysis for the heterogeneous network is shown; the comparison of LPIHN, PRINCE, and RWR in terms of fold enrichment shows that our method is outperforming the other two methods.

The curves of precision and recall of LPIHN, PRINCE, and RWR with the varying threshold 1 ≤ *k* ≤ 96 are shown in Figure 4(a), which shows that LPIHN can achieve the highest precision of 72.1%, while PRINCE and RWR methods achieve lower results with 20.6% and 17.3%, respectively. Meanwhile, compared with PRINCE and RWR, the LPIHN method achieves a higher precision at every recall value. Moreover, the comparison of these methods in terms of average fold enrichment is shown in Figure 4(b). For all of the 96 proteins, LPIHN achieves an average enrichment score of 18.9, which is 10.6% and 10.1% compared to PRINCE and RWR, respectively.

**Figure 5:**
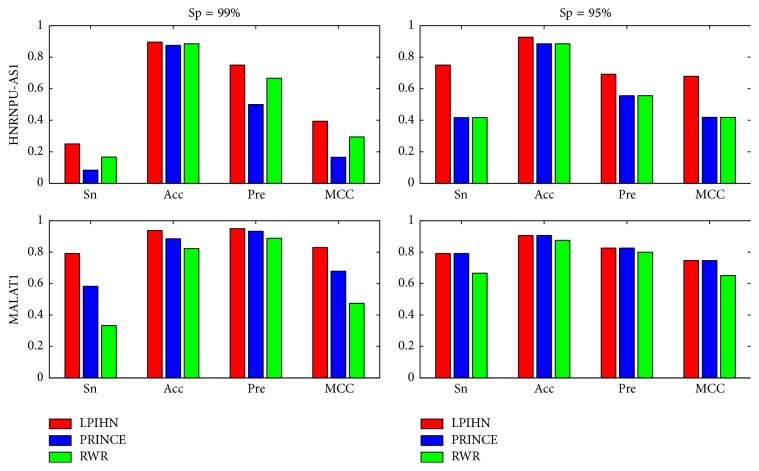
Comparison of three different methods on lncRNA HNRNPU-AS1 and MALAT1 in terms of Sn, Acc, Pre, and MCC. The *x*-axis represents sensitivity, accuracy, precision, and Matthew correlation coefficient, respectively. The left part is at Sp of 99.0% and the right part is at Sp of 95.0%.

**Figure 6:**
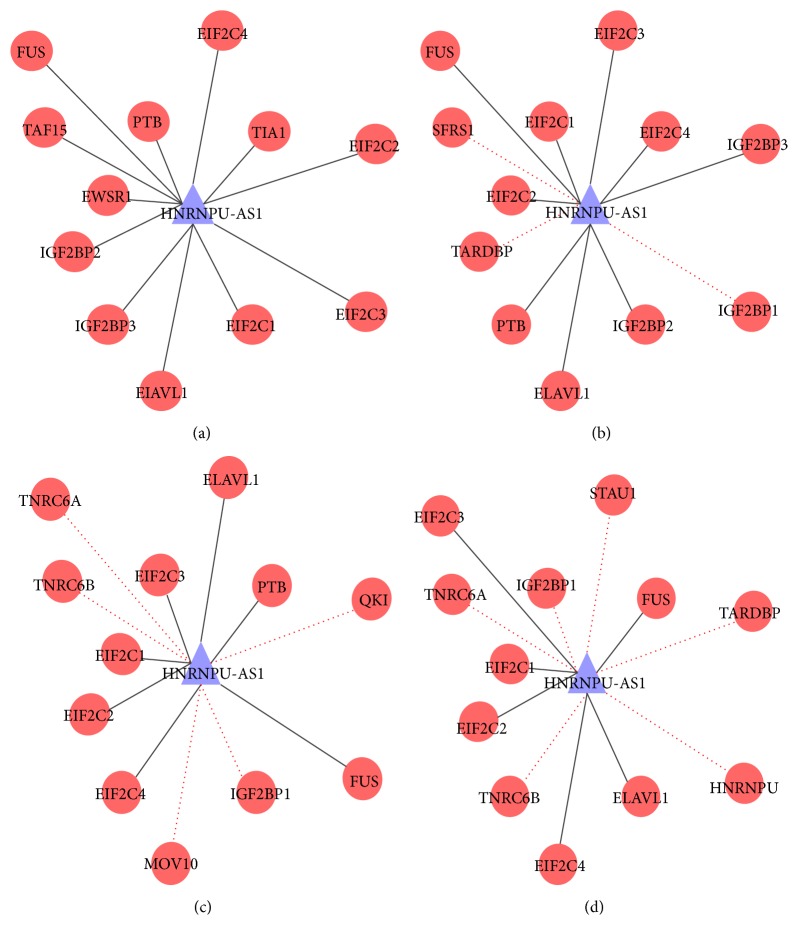
The network of lncRNA HNRNPU-AS1 and the network reconstructed by using LPIHN, PRINCE, and RWR. (a) Known lncRNA-protein interaction network of lncRNA HNRNPU-AS1. (b) Network reconstructed by using LPIHN: solid line indicates known interactions that are correctly predicted and red dotted line new interactions that are not included in known lncRNA-protein interactions. (c) Network reconstructed by using PRINCE. (d) Network reconstructed based on RWR.

To further evaluate the performance of LPIHN, we implement case studies for two lncRNAs including NONHSAT010657 (HNRNPU-AS1) and NONHSAT022127 (MALAT1), which are related to 12 and 24 lncRNA-protein interactions, respectively. The comparison between LPIHN, PRINCE, and RWR in terms of Sn, Acc, Pre, and MCC is shown in Figure 5, which indicates that LPIHN achieves better performance than PRINCE and RWR. In particular, when Sp is 99.0%, for lncRNA HNRNPU-AS1, the Sn, Acc, Pre, and MCC values of LPIHN are increased by 16.7%, 2.1%, 25.0%, and 22.9% when compared with PRINCE, and 7.3%, 1.1%, 8.3%, and 10% when compared with RWR, respectively. For lncRNA MALAT1, the Sn, Acc, Pre, and MCC values of LPIHN are increased by 20.9%, 5.3%, 1.7%, and 25% when compared with PRINCE and 45.9%, 11.5%, 1.7%, and 35.4% when compared with RWR, respectively. Moreover, we reconstruct the interaction network of lncRNA HNRNPU-AS1 by using the prediction data of these three methods (Figure 6). Among the 12 true lncRNA-protein interactions of lncRNA HNRNPU-AS1, LPIHN successfully reconstructs 9 interactions, while PRINCE and RWR retrieve lower interactions of 7 and 6, respectively.

To verify the effect of the number of interactions on the performance of the proposed method, we group the lncRNAs into four equal intervals according to the different number of interactions. Then, AUC values of different intervals are plotted in Figure S2. The result shows that the more the proteins that interact with a query lncRNA are, the better the performance the proposed method can achieve.

### 3.2. Comparison with Existing Methods

**Figure 7:**
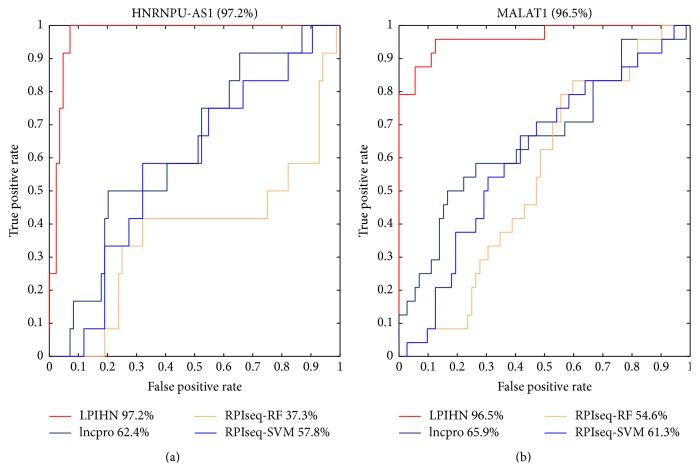
ROC curves and AUC values on lncRNA HNRNPU-AS1 and MALAT1. (a) The ROC curves and AUC values of LPIHN, lncpro, RPIseq-RF, and RPIseq-SVM on lncRNA HNRNPU-AS1. (b) The ROC curves and AUC values on lncRNA MALAT1 by LPIHN, lncpro, RPIseq-RF, and RPIseq-SVM.

We also evaluate the performance of LPIHN on lncRNA HNRNPU-AS1 and MALAT1 with existing methods: lncpro and RPIseq. RPIseq yields two types of scores based on support vector machine (SVM) and random forest (RF), respectively. ROC curves and AUC values of these methods are shown in Figure 7. It is obvious that the ROC curve of LPIHN is consistently above the other methods on both HNRNPU-AS1 and MALAT1. For lncRNA HNRNPU-AS1, the AUC value of LPIHN is 34.8%, 59.9%, and 39.4% higher than lncpro, RPIseq-RF, and RPIseq-SVM, respectively. The AUC value of LPIHN is 30.6%, 41.9%, and 35.2% higher than lncpro, RPIseq-RF, and RPIseq-SVM on MALAT1, respectively. All the evaluations above show that LPIHN outperforms the other two network-based methods and existing methods, which indicates that LPIHN is a powerful method to predict the interactions between lncRNAs and proteins.

### 3.3. Case Studies

**Figure 8:**
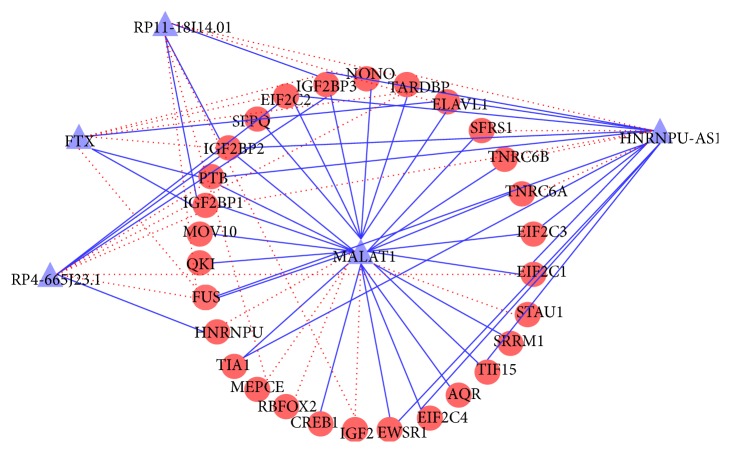
Case study results on lncRNA-protein interaction predictions. Purple and red nodes indicate lncRNAs and proteins, respectively, blue edges known interactions, and red dotted edges newly predicted interactions with 5 highest scores.

The proposed method is able to predict novel lncRNA-protein interactions for the query lncRNA. For each lncRNA, the proteins ranked within top 10 (this is a user-defined threshold) are considered as the potential proteins interacting with the query lncRNA. To further evaluate the efficiency of LPIHN to predict novel lncRNA-protein interactions, we present case studies of five lncRNAs, including NONHSAT137627 (FTX), HNRNPU-AS1, MALAT1, NONHSAT004412 (RP4-665J23.1), and NONHSAT016118 (RP11-18I14.10).  Figure 8 shows the predicted network for these lncRNAs, where the known lncRNA-protein interactions and top 5 ranked predictions are displayed. For lncRNA FTX, HNRNPU-AS1, and MALAT1, the top 10 predictive proteins are listed in Table 2 (Table S1 for lncRNA RP4-665J23.1 and RP11-18I14.10). In the prediction result, 9606.ENSP00000258729 (IGF2BP3), 9606.ENSP00000254108 (FUS), 9606.ENSP00000371634 (IGF2BP2), 9606.ENSP00000401371 (TIA1), and 9606.ENSP00000258962 (SFRS1) are predicted to interact with FTX. 9606.ENSP00000290341 (IGF2BP1) and SFRS1 are predicted to interact with HNRNPU-AS1. FUS are predicted to interact with RP4-665J23.1. 9606.ENSP00000349428 (PTB) and FUS are predicted to interact with RP11-18I14.10. The predictions above are all confirmed by starBase, a database for known protein-RNA and miRNA-target interactions [36]. In our prediction result, 9606.ENSP00000283179 (HNRNPU) is predicted to interact with MALAT1, which is confirmed by the research done by Xiao et al. [37]. Moreover, the top 3 ranked proteins of lncRNA FTX in our study are IGF2BP3, FUS, and IGF2BP2. For the above predictions confirmed by evidence in research or database, we compare their ranks by LPIHN, PRINCE, and RWR (Table S2), which shows that LPIHN achieves a higher rank of almost every candidate protein. This further indicates the efficiency of our method to predict novel proteins for lncRNAs.

## 4. Conclusion

With the development of the research of lncRNA, computational methods have been published for the predictions of lncRNA-protein interactions. In this paper, we introduce a network-based method LPIHN to predict the proteins interacting with lncRNAs. First, a heterogeneous network is constructed by connecting PPI and lncRNA-lncRNA similarity network using known lncRNA-protein interactions. Then, an iteratively random walk is implemented on the heterogeneous network, which can score proteins for each lncRNA. Finally, LOOCV is implemented to evaluate the performance of our method. The results show that LPIHN obtains an AUC of 96.0%, which is much higher than PRINCE and RWR. Moreover, when focusing on the top 2% (4870) predicted lncRNA-protein interactions, LPIHN successfully reconstructs 802 interactions, while PRINCE and RWR retrieve much lower interactions of 229 and 192, respectively. Meanwhile, the other measures also show that LPIHN algorithm outperforms PRINCE and RWR method, which propagate information only in protein network. We also demonstrate the efficiency of LPIHN to predict novel lncRNA-protein interactions; some top-ranked lncRNA-protein interactions predicted by our method are supported by existing literature or database. The good performance and the practical value show that our approach is a promising way to predict potential lncRNA-protein interactions.

While the results are promising, the LPIHN method shows some limitations. Firstly, we test our method only on one database (i.e., NPinter 2.0). From the known lncRNA-protein interaction dataset, we observe that each lncRNA interacts with about 4.37 proteins on average. Due to the relative sparsity of the known lncRNA-protein interactions, the network-based method may produce biased predictions. This situation can be improved by the increase of comprehensive lncRNA-protein interactions datasets. Secondly, skewed degree distribution of the network may affect the result of our prediction; adding some appropriate resistance in the process of random walk may improve the performance of our method. Thirdly, the proposed method can only predict similarity between lncRNAs that have expression profile, which indicates that the increase of lncRNA-protein interaction datasets may lead to the incomplete coverage of the lncRNA-lncRNA similarity network. This situation can be improved by adding information such as known lncRNA-protein interactions.

## Supplementary Material

Figure S1 -The ROC curve and AUC value LPIHN, PRINCE and RWR on the new dataset.
Figure S2 - Comparsion of AUC by LPIHN on different intervals .
lncRNAs are grouped into four equal intervals according to the different number of interactions. Then, AUC values of different intervals are displayed.
Table S1 - The top 10 ranked proteins for lncRNA RP4-665J23.1 and RP11-18I14.10.
Table S2 - Top candidate proteins predicted by LPIHN with reference support and their ranks predicted by PRINCE and RWR.

## Figures and Tables

**Table 1:** Comparison of three different methods in terms of AUC, Sn and Sp.

	LPIHN	PRINCE	RWR
AUC	96.0%	90.6%	88.1%
Sn	35.0%	5.0%	14.1%
Sp	99.0%		
Sn	73.1%	26.7%	35.3%
Sp	95.0%		
Sn	91.4%	55.1%	59.2%
Sp	90.0%		

**Table 2:** The top 10 ranked proteins for lncRNA FTX, HNRNPU-AS1 and MALAT1.

Gene	String ID	Rank	Gene	String ID	Rank
FTX (NONCODE ID: NONHSAT137627)					
IGF2BP3	9606.ENSP00000258729	1	TIA1	9606.ENSP00000401371	6
FUS	9606.ENSP00000254108	2	SFRS1	9606.ENSP00000258962	7
IGF2BP2	9606.ENSP00000371634	3	RBFOX2	9606.ENSP00000413035	8
TARDBP	9606.ENSP00000240185	4	EIF2C1	9606.ENSP00000362300	9
EIF2C2	9606.ENSP00000220592	5	QKI	9606.ENSP00000354951	10

HNRNPU-AS1 (NONCODE ID: NONHSAT010657)					
IGF2BP1	9606.ENSP00000290341	1	MOV10	9606.ENSP00000350028	6
TARDBP	9606.ENSP00000240185	2	IGF2	9606.ENSP00000338297	7
SFRS1	9606.ENSP00000258962	3	HNRNPU	9606.ENSP00000283179	8
TNRC6B	9606.ENSP00000338371	4	STAU1	9606.ENSP00000360922	9
TNRC6A	9606.ENSP00000379144	5	SFPQ	9606.ENSP00000349748	10

MALAT1 (NONCODE ID: NONHSAT022127)					
HNRNPU	9606.ENSP00000283179	1	CDK9	9606.ENSP00000362361	6
RBFOX2	9606.ENSP00000413035	2	CTCF	9606.ENSP00000264010	7
IGF2	9606.ENSP00000338297	3	NXF1	9606.ENSP00000294172	8
STAU1	9606.ENSP00000360922	4	LRRK2	9606.ENSP00000298910	9
MEPCE	9606.ENSP00000308546	5	SSB	9606.ENSP00000260956	10
