# Supplementary material for: Predicting Long Noncoding RNA and Protein Interactions Using Heterogeneous Network Model
Source: Biomed Res Int. 2015 Dec 29;2015:671950. doi: 10.1155/2015/671950 (PMC4709602; doi:10.1155/2015/671950)
Supplement: Supplementary file 1 — Figure S1 -The ROC curve and AUC value LPIHN, PRINCE and RWR on the new dataset. Figure S2 - Comparsion of AUC by LPIHN on different intervals . lncRNAs are grouped into four equal intervals according to the different number of interactions. Then, AUC values of different intervals are displayed. Table S1 - The top 10 ranked proteins for lncRNA RP4-665J23.1 and RP11-18I14.10. Table S2 - Top candidate proteins predicted by LPIHN with reference support and their ranks predicted by PRINCE and RWR. [file 671950.f1.docx]

**Figure S1 –The ROC curve and AUC value LPIHN, PRINCE and RWR on the new dataset**

**
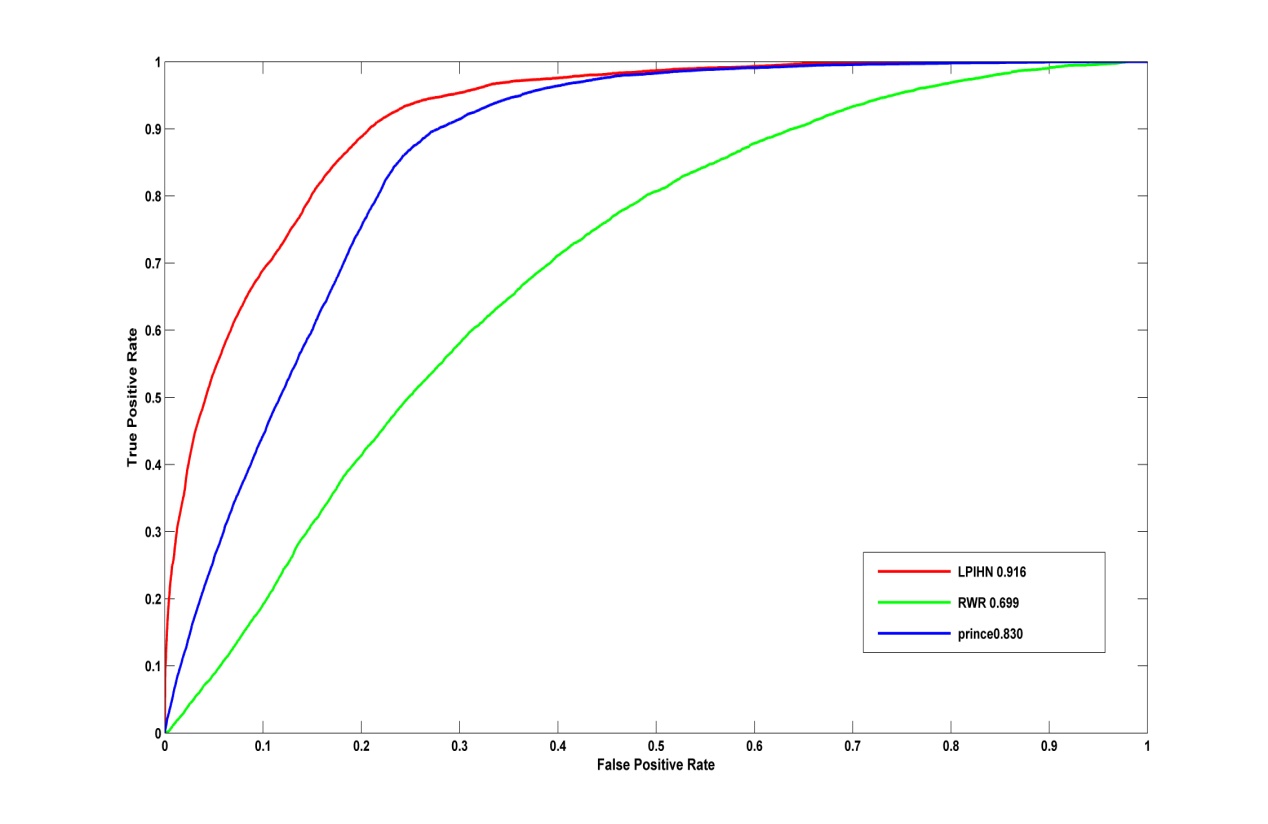
**

**Figure S2- Comparsion of AUC by LPIHN on different intervals**

the lncRNAs are grouped into four equal intervals according to the different number of interactions. Then, AUC values of different intervals are displayed.


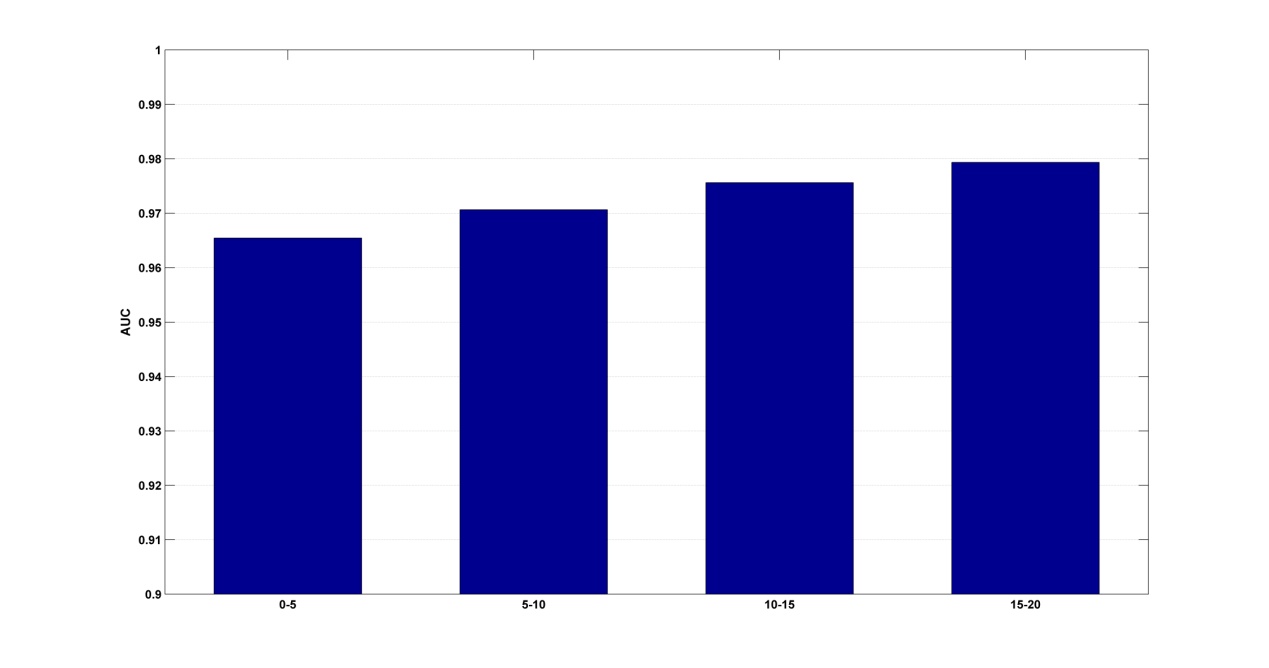


**Table S1 – The top 10 ranked proteins for lncRNA RP4-665J23.1 and RP11-18I14.10**

| **RP4-665J23.1 (NONCODE ID: NONHSAT004412)** | | | | | |
| --- | --- | --- | --- | --- | --- |
| Gene | String ID | Rank | Gene | String ID | Rank |
| IGF2BP1 | 9606.ENSP00000290341 | 1 | IGF2 | 9606.ENSP00000338297 | 6 |
| FUS | 9606.ENSP00000254108 | 2 | SFRS1 | 9606.ENSP00000258962 | 7 |
| TARDBP | 9606.ENSP00000240185 | 3 | MEPCE | 9606.ENSP00000308546 | 8 |
| PTB | 9606.ENSP00000349428 | 4 | MOV10 | 9606.ENSP00000350028 | 9 |
| EIF2C1 | 9606.ENSP00000362300 | 5 | EIF2C4 | 9606.ENSP00000362306 | 10 |
| **RP11-18I14.10 (NONCODE ID: NONHSAT016118)** | | | | | |
| Gene | String ID | Rank | Gene | String ID | Rank |
| ELAVL1 | 9606.ENSP00000385269 | 1 | EIF2C2 | 9606.ENSP00000220592 | 6 |
| FUS | 9606.ENSP00000254108 | 2 | MOV10 | 9606.ENSP00000350028 | 7 |
| IGF2 | 9606.ENSP00000338297 | 3 | PTB | 9606.ENSP00000349428 | 8 |
| TARDBP | 9606.ENSP00000240185 | 4 | EIF2C1 | 9606.ENSP00000362300 | 9 |
| MEPCE | 9606.ENSP00000308546 | 5 | CTCF | 9606.ENSP00000264010 | 10 |

**Table S2 - Top candidate proteins predicted by LPIHN with reference support and their ranks predicted by PRINCE and RWR.**

| **FTX** | | | |
| --- | --- | --- | --- |
| Gene | Rank by LPIHN | Rank by PRINCE | Rank by RWR |
| IGF2BP3 | 1 | 13 | 14 |
| FUS | 2 | 6 | 6 |
| IGF2BP2 | 3 | 17 | 21 |
| TIA1 | 6 | 14 | 10 |
| SFRS1 | 7 | 7 | 7 |
| **HNRNPU-AS1** | | | |
| Gene | Rank by LPIHN | Rank by PRINCE | Rank by RWR |
| IGF2BP1 | 1 | 4 | 3 |
| SFRS1 | 3 | 7 | 8 |
| **MALAT1** | | | |
| Gene | Rank by LPIHN | Rank by PRINCE | Rank by RWR |
| HNRNPU | 1 | 3 | 1 |
| **RP4-665J23.1** | | | |
| Gene | Rank by LPIHN | Rank by PRINCE | Rank by RWR |
| FUS | 2 | 7 | 11 |
| **RP11-18I14.10** | | | |
| Gene | Rank by LPIHN | Rank by PRINCE | Rank by RWR |
| FUS | 2 | 11 | 7 |
| PTB | 8 | 8 | 19 |
